# Integrating Wound Images and Clinical Text for Pressure Injury Assessment and Treatment Recommendation

**DOI:** 10.3390/bioengineering13060642

**Published:** 2026-05-29

**Authors:** Binyang Wang, Yuxue Wang, Tong Sun, Yingke Chen, Liangchen Liu, Chuanxiong Li, Jin Yan

**Affiliations:** 1 Kunming Medical University, Kunming 650500, China; 20201284@kmmu.edu.cn (B.W.); wangyuxue@kmmu.edu.cn (Y.W.); 20232018@kmmu.edu.cn (T.S.); lichuanxiong@kmmu.edu.cn (C.L.); 2 Yunnan University, Kunming 650500, China; 3 Northumbria University, Newcastle Upon Tyne NE1 8ST, UK; yingke.chen@northumbria.ac.uk; 4 Emory University, Atlanta, GA 30322, USA; liangchen.liu@emory.edu; 5 Columbia University, New York, NY 10027, USA

**Keywords:** pressure injury, multimodality, large language model, clinical decision support, multimodal

## Abstract

Pressure injury management requires coordinated decisions on wound staging, debridement, and dressing selection, yet most computational studies focus on isolated tasks using a single data modality. This study developed a multimodal, multi-task framework integrating wound photographs and clinical text to support pressure injury stage classification, debridement necessity prediction, primary dressing recommendation, and secondary dressing recommendation. Models were trained on an expert-annotated public dataset and externally validated using an independent private cohort. We systematically compared image-only models, text-only models, large language model-based approaches, conventional image–text fusion strategies, and image–language model combinations. Among the evaluated models, ResNet-50 combined with DeepSeek-R1-Distill-Qwen-1.5B showed the best overall balance across classification performance, text-generation quality, and validation robustness. On the validation set, the final model achieved 82.92% accuracy, 81.26% macro-F1, and 99.01% area under the curve for stage classification, and 89.91% accuracy, 89.83% macro-F1, and 96.24% area under the curve for debridement prediction. For dressing recommendation, the model achieved ROUGE-1 scores of 58.30% and 61.06% for primary and secondary dressings, respectively. Interpretability analyses indicated clinically relevant image attention and text attribution patterns. These findings suggest that multimodal learning may provide a clinically aligned decision-support approach for pressure injury assessment and treatment recommendations.

## 1. Introduction

Pressure injuries (PIs), also known as pressure ulcers or decubitus ulcers, are among the most common and clinically important adverse events in healthcare settings worldwide [1]. They are localized injuries to the skin and underlying soft tissue, typically caused by sustained mechanical loading over bony prominences, and are particularly common among patients with limited mobility, including those in intensive care units, long-term care facilities, and rehabilitation wards [2,3]. A large-scale meta-analysis including more than 2.5 million hospitalized adults reported a global PI prevalence of approximately 12.8%, while the annual cost of PI management has been estimated to reach billions of dollars, even in high-income countries [4,5]. Beyond this economic burden, PIs are associated with increased morbidity, prolonged hospital stays, infection risk, and reduced quality of life, highlighting the need for accurate and timely wound assessment.

Clinical management of PIs requires a sequence of related decisions, including wound staging, assessment of debridement necessity, and selection of appropriate dressings [6]. In routine practice, these decisions still rely heavily on clinical experience and subjective judgment, which may lead to variability across clinicians and institutions. Recent advances in artificial intelligence (AI) and deep learning have therefore encouraged the development of automated decision-support tools for wound assessment and management.

Over the past several years, studies have shown the feasibility of applying convolutional neural networks (CNNs), including ResNet, VGGNet, DenseNet, and YOLO-based models, to PI stage classification using wound photographs [7,8,9,10,11,12]. These image-based approaches have achieved promising performance, in some cases approaching the accuracy of trained wound care nurses. More recently, Vision Transformers (ViTs) have also been explored for wound image analysis, as their self-attention mechanisms can capture global spatial relationships beyond local convolutional features [13]. In parallel, large language models (LLMs) have enabled clinical text to be incorporated into wound assessment through multimodal architectures or parameter-efficient fine-tuning strategies such as Low-Rank Adaptation (LoRA) [14].

Despite these advances, several challenges remain. First, most existing models focus on isolated tasks, particularly PI staging, while downstream decisions such as debridement assessment and dressing selection remain underexplored. Second, many studies rely primarily on wound images as the sole input [8,9]. In real-world practice, however, wound assessment depends not only on visual appearance but also on clinical descriptions, including tissue characteristics, exudate, wound depth, and surrounding skin condition [15]. Although some studies have incorporated basic clinical metadata for general wound classification [16,17], fine-grained multimodal systems designed specifically for the PI management pathway remain limited. Third, many reported models have not been evaluated using independent external cohorts, leaving their generalizability across clinical settings uncertain.

To address these limitations, we developed a multimodal, multi-task deep learning framework for PI assessment and treatment recommendation. By integrating wound photographs with clinical descriptions, the proposed framework jointly predicts four clinically relevant outputs: PI stage, debridement necessity, primary dressing selection, and secondary dressing selection. To identify an effective modelling strategy, we systematically benchmarked a range of architectures, from conventional unimodal networks to multimodal fusion models and vision–language model combinations. The framework was further evaluated using both an expert-annotated public dataset and an independent external cohort, providing a more rigorous assessment of its potential clinical generalizability.

## 2. Related Works

Traditional pressure injury assessment relies on structured clinical criteria and wound scoring tools to evaluate wound stage, tissue characteristics, exudate, and healing status. Although these tools support standardized documentation and longitudinal monitoring, their use remains dependent on clinician experience and subjective interpretation. This limitation is clinically relevant because pressure injury management involves a sequence of related decisions, including staging, debridement assessment, and dressing selection [6].

Deep learning has been increasingly applied to automated wound image analysis. Convolutional neural networks (CNNs), including ResNet, VGGNet, DenseNet, and YOLO-based models, have been explored for pressure injury detection and stage classification from wound photographs [7,8,9,10,11,12]. More recently, Mohan et al. applied a Vision Transformer model to diabetic foot ulcer detection, suggesting that transformer-based visual models may capture wound-related features beyond conventional convolutional representations [18]. These studies support the value of image-based learning for wound assessment. However, visual models alone may not fully capture clinical context, such as exudate characteristics, tissue descriptions, wound history, infection status, and treatment requirements.

Clinical text and language models provide another important source of wound-related information. Structured or semi-structured wound descriptions often document clinically relevant features, including tissue type, necrosis, exudate, wound edge, surrounding skin condition, and treatment needs, all of which are closely related to staging, debridement assessment, and dressing selection [6,19]. Meanwhile, clinical natural language processing studies have shown that neural text encoders and large language models can extract meaningful information from medical text and support clinical prediction or decision-making tasks [20,21]. Nevertheless, text-only models cannot directly observe wound morphology and may be insufficient when decisions depend on visual evidence, such as wound color, necrotic burden, tissue coverage, wound depth, and periwound condition. These limitations provide a rationale for multimodal wound-care models that integrate clinical text with image-derived information [17,22].

Multimodal learning has therefore gained increasing attention in wound care. Nguyen et al. combined wound-related visual features with clinical text to support actionable wound care decisions, showing that textual descriptions can complement image-derived information [23]. More recently, Fard et al. proposed a deep multimodal wound assessment tool that integrates wound images and clinical notes for home wound referral decision-making [17]. These studies demonstrate the potential of image–text fusion for wound decision support. However, most existing multimodal models focus on referral recommendation, wound typing, or other single endpoints, rather than the broader pressure injury management pathway.

In contrast, our study develops a multimodal framework for four clinically connected pressure injury tasks: stage classification, debridement necessity prediction, primary dressing selection, and secondary dressing selection. Rather than focusing on a single endpoint, we systematically benchmark image-only, text-only, conventional image–text fusion, and image–language model fusion strategies within a unified multi-task setting. This design extends previous wound assessment studies from isolated prediction tasks toward a more comprehensive decision-support pathway for pressure injury care.

## 3. Materials and Methods

### 3.1. Problem Definition and Dataset

#### 3.1.1. Dataset Selection and De-Duplicated Images

**Figure 1:**
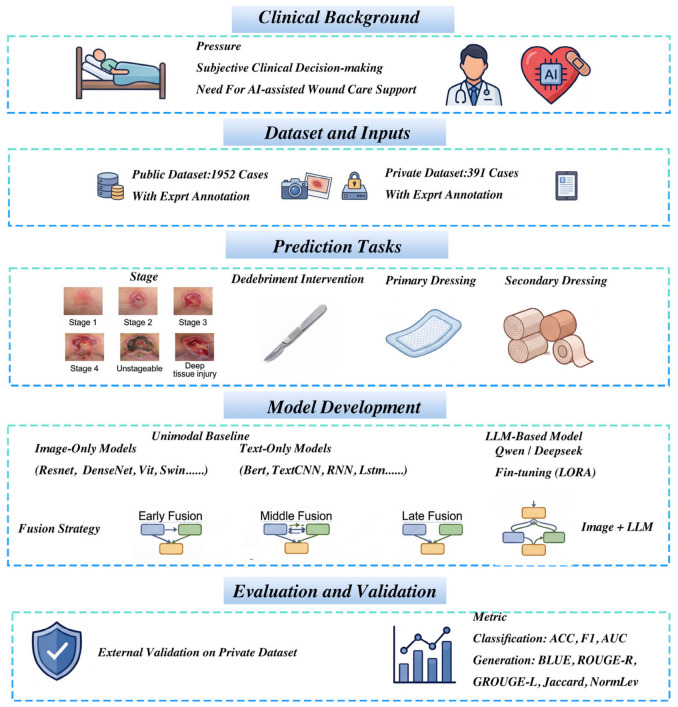
Overview of the study design and multimodal pressure injury assessment framework. The figure illustrates the overall workflow, including dataset construction, prediction tasks, model development, multimodal fusion strategies, and external validation for pressure injury assessment and treatment recommendation.

This study utilized two distinct wound care datasets for model development and external validation. The overall data curation and annotation workflow is illustrated in Figure 1.

Public Dataset (PISTA). We assembled the Pressure Injury Staging and Treatment Annotation (PISTA) dataset by aggregating open-access wound photographs from the Roboflow platform and GitHub repositories. Following data cleaning and deduplication, the finalized dataset comprised 2105 unique images. Certified wound care nurses independently annotated each image for PI staging and related clinical characteristics according to established guidelines. Any annotation discrepancies were effectively resolved through expert consensus.

Private Dataset. To assess cross-center generalizability, an independent external cohort of 391 cases was obtained from the Affiliated Hospital of Yunnan University (Ethics Approval ID 2026-52). Acquired using different imaging equipment and annotated by a distinct group of clinicians, this dataset introduces realistic distribution shifts in both visual appearance and textual documentation styles. Wound images were generally acquired after removal of covering dressings, with the camera or smartphone positioned above the wound under adequate lighting and focus. When feasible, the image field included the complete wound boundary and adjacent surrounding skin to preserve contextual information.

#### 3.1.2. Annotation Protocol and Principles

All images were annotated according to international pressure injury guidelines and contemporary wound care concepts, including wound bed preparation, debridement and dressing selection [24]. Each image was independently annotated across three clinical dimensions: pressure injury stage, wound bed tissue type, and dressing recommendation. Pressure injury staging followed the NPUAP/EPUAP/PPPIA classification system [25], with categories including Stages 1–4, Unstageable, and Deep Tissue Injury (DTI). Wound bed tissue type was labeled using the Red–Yellow–Black (RYB) framework, with the primary label assigned according to the predominant tissue type visible in the photograph. Dressing annotations included a primary dressing (assigned for all images) and, where applicable, a secondary dressing, with permissible dressing types defined according to stage-based clinical guidelines.

All annotations were performed by a panel of four certified wound care nurses and three clinicians with expertise in pressure injury assessment. Prior to annotation, the team collaboratively developed a written guideline specifying labeling criteria for each dimension, which has been made publicly available in our GitHub repository. Images presenting ambiguity were resolved through group discussion; when consensus could not be reached by discussion alone, a majority-vote procedure was applied. Annotators were instructed to base all labels solely on photographic appearance, supplemented by minimal contextual information where available.

Each wound item was paired with one corresponding clinical text annotation and structured labels for pressure injury stage, debridement requirement, primary dressing recommendation, and secondary dressing recommendation. To illustrate the annotation format and clinical content, eight representative examples of text annotations and corresponding structured labels are provided in Supplementary Table S11.

#### 3.1.3. Task Definition

We formulate wound care decision support as a multi-task prediction problem comprising four concurrent tasks. Wound staging is a six-class classification task that assigns each wound to one of the NPIAP-defined categories: Stage I, II, III, IV, Unstageable, or Suspected DTI. Debridement decision is a binary classification task determining whether debridement intervention is indicated. Primary and secondary dressing recommendation is a short phrase selecting the most appropriate contact-layer dressing from: hydrogel, silver-containing, alginate, foam, hydrocolloid, or other. All labels were assigned by three certified wound care nurses and three clinicians through the structured consensus process.

### 3.2. Multimodal Feature Encoders

#### 3.2.1. Image Encoder

Six representative image encoders were evaluated to extract visual features from wound photographs, including ResNet-18, ResNet-50, DenseNet-121, MobileNetV2, ViT-B/16, and Swin-T. All images were resized to 224 × 224 pixels and normalized using the ImageNet mean and standard deviation. During training, standard data augmentation was applied, including random horizontal flipping, random cropping, and color jittering. For each encoder, the output of the final global average pooling layer was used as the image feature representation and then projected into a shared embedding space by a learnable linear layer.

#### 3.2.2. Text Encoder

Four text encoders were evaluated to process the clinical descriptions paired with each wound image: BERT, TextCNN, bidirectional RNN, and bidirectional LSTM. For BERT, subword tokenization was applied, and sequences were truncated or padded to a maximum length of 512 tokens. For TextCNN, RNN, and LSTM, Chinese word segmentation was performed using the Jieba toolkit, and sequences were limited to 256 tokens. The output of each text encoder was used as the text feature representation and projected into the same shared embedding space as the image features.

### 3.3. Multimodal Fusion Strategies

To investigate how visual and textual information should be integrated, we compared three multimodal fusion strategies: early fusion, middle fusion, and late fusion.

#### 3.3.1. Early Fusion (Feature Concatenation)

In the early fusion strategy, the projected image and text feature vectors are concatenated along the feature dimension to form a joint multimodal representation:

f_early = [f^^I^;f^^T^] ∈ ℝ^2D^

The concatenated feature was then passed through a shared multilayer perceptron with ReLU activation and dropout to obtain the fused representation for downstream prediction.

#### 3.3.2. Middle Fusion (Cross-Attention Interaction)

In the middle fusion setting, cross-modal interaction was modeled using a multi-head cross-attention module. The text feature served as the query, whereas the image feature served as the key and value. The attention output was combined with the original text representation through residual connection and layer normalization, followed by a feed-forward network to obtain the fused representation. This strategy was designed to explicitly align textual clinical attributes with corresponding visual evidence.

#### 3.3.3. Late Fusion (Decision-Level Fusion)

In the late fusion setting, the image and text branches were modeled independently, and each branch generated task-specific prediction probabilities. The final prediction was obtained by averaging the two probability distributions with equal weights:

P_late = α · P^I^ + (1 − α) · P^T^, α = 0.5

This strategy preserves modality-specific decision boundaries while combining the two modalities at the output level.

### 3.4. Large Language Model Fine-Tuning

In addition to conventional encoder-based pipelines, we evaluated large language model (LLM)-based approaches for text-driven wound assessment and recommendation. For text-only LLMs, Low-Rank Adaptation (LoRA) was used for parameter-efficient fine-tuning. LoRA inserts trainable low-rank matrices into selected attention projections while keeping the original pretrained parameters frozen. In this study, LoRA modules were applied to the query and value projections of the self-attention layers, with rank r = 8 and scaling factor α = 16.

Because text-only LLMs cannot directly process images, each sample was represented by its clinical wound description and a standardized task prompt. The prompt was designed to constrain the model output into a predefined JSON-like structure containing four fields: pressure injury stage, debridement decision, primary dressing recommendation, and secondary dressing recommendation. The generated outputs were subsequently parsed into structured labels for quantitative evaluation. For reproducibility, the same prompt format and label definitions were used across comparable LLM experiments.

Fine-tuning was performed using the AdamW optimizer with a learning rate of 2 × 10^−5^, batch size of 3, and 5 training epochs. During inference, decoding parameters were fixed within each experimental setting. API-based LLM classification used a low temperature setting of 0.1 and a maximum output length of 256 tokens to improve output determinism. LoRA fine-tuned models used a maximum output length of 512 tokens, a temperature of 0.7, top-p of 0.9, and sampling-based decoding. Complete prompt templates, decoding parameters, token settings, model specifications, and reproducibility details are provided in the Supplementary File S1.

### 3.5. Multi-Task Learning Framework

All models were trained under a unified multi-task framework. Four task-specific heads were attached to the final representation, corresponding to Stage, Clean, Primary, and Secondary tasks. For **encoder**-based models, predictions were generated from the fused feature representation. For LLM-based models, task outputs were derived from the generated structured response.

For classification tasks, each head was implemented as an independent fully connected layer followed by softmax:

ŷ_k_ = softmax (W_k_h + b_k_), k ∈ {Stage, Clean, Primary, Secondary}

where h denotes the final model representation.

The overall training objective was defined as the sum of task-specific cross-entropy losses:

ℒ = ∑_k_ ℒ_k_^ce^

All tasks were equally weighted during training.

### 3.6. Evaluation Metrics

To address class imbalance during model training, particularly for underrepresented pressure injury stages such as deep tissue injury and for debridement prediction, a class-weighted loss strategy was used for the classification tasks in the stability experiments. For each task, the weight of class i was calculated as $w_{i}=N/(K\times N_{i})$, where N is the total number of samples, K is the number of classes, and $N_{i}$ is the number of samples in class i. The weights were then normalized so that $\sum_{i}w_{i}=K$. This weighting scheme increased the relative contribution of minority classes to the training loss and reduced the dominance of majority classes during optimization. In addition, macro-averaged F1 score was used as the primary evaluation metric, and all F1 values reported in this study refer to macro-F1, which gives equal importance to each class and is more sensitive to minority-class performance under imbalanced data distributions.

For expert comparison, three specialist nurses with different experience levels independently evaluated a representative subset of 20 pressure injury photographs while blinded to the model predictions. Inter-expert agreement was calculated as the proportion of cases in which all three experts agreed, two experts agreed while one disagreed, or all three experts provided different conclusions.

For classification tasks, accuracy serves as the primary metric, with precision, recall, and macro-averaged F1 score reported as complementary measures to account for per-class performance and label imbalance. For the two dressing recommendation tasks, which are framed as text generation, BLEU and ROUGE-L scores evaluate lexical overlap and longest common subsequence similarity between generated and reference texts, respectively, while METEOR is additionally reported to accommodate synonym variation and stemming, mitigating the bias of strict string matching in clinical terminology.

## 4. Results

As shown in Figure 1, the overall workflow of this study followed a stepwise path from unimodal to multimodal modeling, including dataset construction, unimodal baseline evaluation, multimodal model screening, and subsequent analysis of the optimal model.

### 4.1. Study Population and Dataset Characteristics

**Figure 2:**
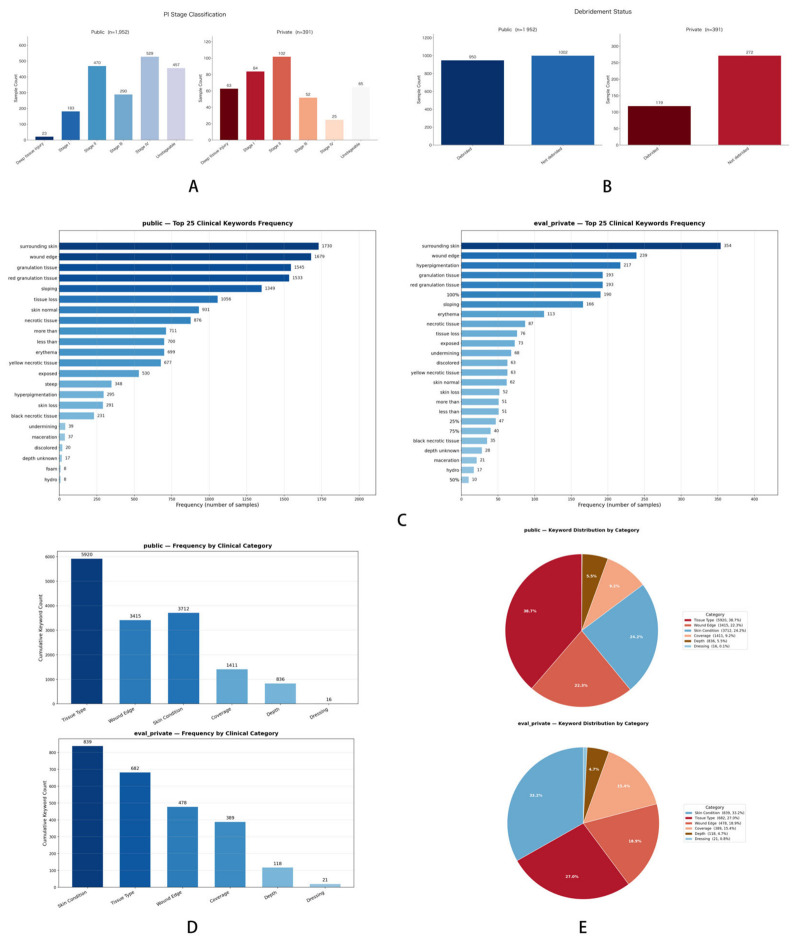
Study population and dataset characteristics. (**A**) Class distribution of the pressure injury stage classification task in the public and private evaluation datasets; (**B**) Class distribution of the debridement status task in the public and private evaluation datasets; (**C**) Top 25 most frequent clinical keywords in the public and private evaluation datasets; (**D**) Frequency distribution of different clinical keyword categories in the public and private evaluation datasets; (**E**) Proportional composition of different clinical keyword categories in the public and private evaluation datasets.

A total of 1952 public cases and 391 private evaluation cases were included in this study. For the pressure injury stage classification task, the public dataset showed an imbalanced class distribution, mainly consisting of Stage IV, Stage II, and Unstageable cases, whereas Deep tissue injury was rare. In contrast, the private evaluation dataset was dominated by Stage II and Stage I cases, with markedly fewer Stage IV cases, indicating a clear distributional difference between the two datasets (Figure 2A). For the debridement status task, the public dataset was relatively balanced between debrided and not debrided samples, whereas the private evaluation dataset was skewed toward not debrided cases, indicating a more pronounced class imbalance (Figure 2B).

Clinical keyword analysis showed that both datasets were dominated by terms related to local wound description, with “surrounding skin” and “wound edge” being the most frequent keywords (Figure 2C). The public dataset placed greater emphasis on tissue type and wound-edge morphology, whereas the private evaluation dataset contained relatively more terms describing skin condition and quantitative proportions (Figure 2C). Category-level analysis further showed that keywords in the public dataset were mainly concentrated in Tissue type, Skin condition, and Wound edge, whereas Skin condition was the most frequent category in the private evaluation dataset (Figure 2D,E). Overall, the public and private datasets differed substantially in both label distribution and clinical text composition, suggesting the presence of class imbalance and distribution shift that should be considered during model development and evaluation.

### 4.2. Results of Image-Only Models

**Figure 3:**
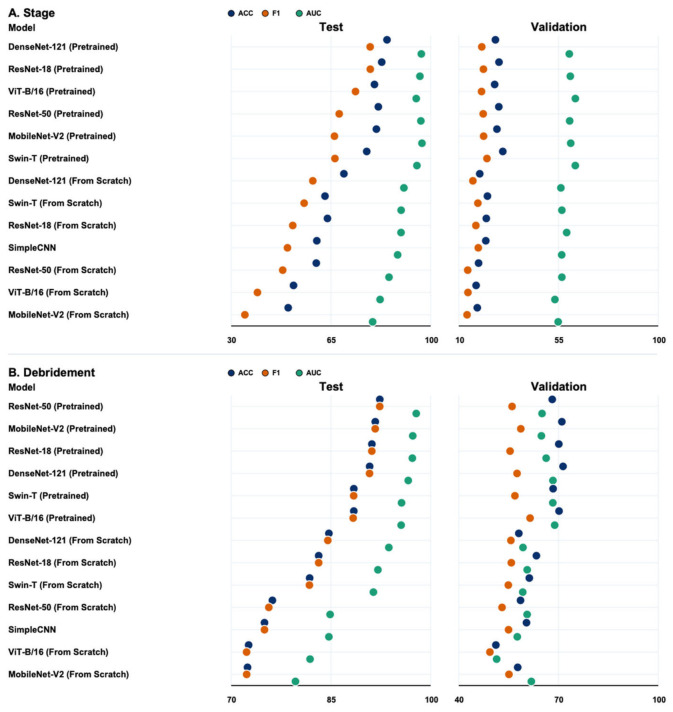
Left-right dot plots of image-only model performance for stage and debridement classification in the test and validation sets. Accuracy (ACC), F1-score (F1), and area under the receiver operating characteristic curve (AUC) are shown.

In the image-only experiments (Table S1), in Figure 3, 13 convolutional neural network and vision transformer architectures were evaluated, including SimpleCNN, ResNet-18, ResNet-50, DenseNet-121, MobileNet-V2, ViT-B/16, and Swin-T, with pretrained and from-scratch variants when applicable. Overall, pretrained models consistently outperformed their from-scratch counterparts in both classification tasks. On the test set, image-only models performed better for Debridement Intervention classification than for Stage classification. For Stage classification, ImageNet-pretrained DenseNet-121 achieved the highest accuracy (84.69% ± 6.75%) and F1-score (78.77% ± 6.16%), while ImageNet-pretrained MobileNet-V2 achieved the highest AUC (96.95% ± 1.61%). For Debridement Intervention classification, ImageNet-pretrained ResNet-50 achieved the best overall performance, with an accuracy of 92.35% ± 4.87%, an F1-score of 92.34% ± 4.87%, and an AUC of 97.83% ± 2.05%. In contrast, performance declined markedly on the validation set. For Stage classification, accuracy ranged from 17.82% to 29.84% and AUC from 53.38% to 62.61%; for Debridement Intervention classification, accuracy ranged from 51.15% to 71.36% and AUC from 51.39% to 68.85%. These results indicate that although pretrained image encoders captured clinically relevant wound features effectively, their generalizability to unseen data remained limited, particularly for Stage classification.

### 4.3. Results of Text-Only Models

**Figure 4:**
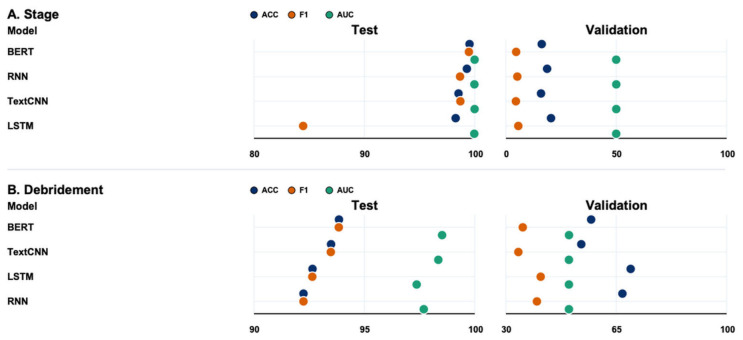
Left-right dot plots of conventional text-only model performance for stage and debridement classification in the test and validation sets. ACC, F1, and AUC are shown.

**Figure 5:**
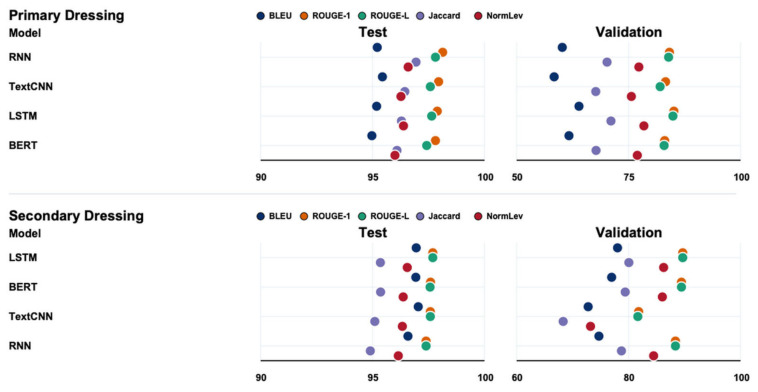
Left-right dot plots of conventional text-only model performance for text generation in the test and validation sets. BLEU, ROUGE-1, ROUGE-L, Jaccard similarity, and normalized Levenshtein similarity (NormLev) are shown.

In the text-only experiments, conventional text encoders and large language models showed distinct performance patterns (Table S2), in Figure 4. Among the conventional text encoders, BERT achieved the best overall classification performance on the test set, reaching 99.53% ± 0.55% accuracy, 99.47% ± 0.87% F1-score, and 99.99% ± 0.03% AUC for Stage classification, and 93.85% ± 0.58% accuracy, 93.84% ± 0.58% F1-score, and 98.52% ± 0.38% AUC for Debridement Intervention classification. However, the performance of conventional text-only models dropped markedly on the validation set, with Stage classification accuracies of only 15.91–20.43% and AUC values close to 50%, indicating limited generalizability. For text generation (Table S3), in Figure 5, BERT achieved the best overall performance on the Public Test set, whereas LSTM showed the strongest and most stable results on the more challenging Private Eval set. Overall, conventional text-only models were effective in capturing clinically relevant semantic information, with BERT serving as the strongest classification baseline and LSTM showing greater robustness in generation tasks.

**Figure 6:**
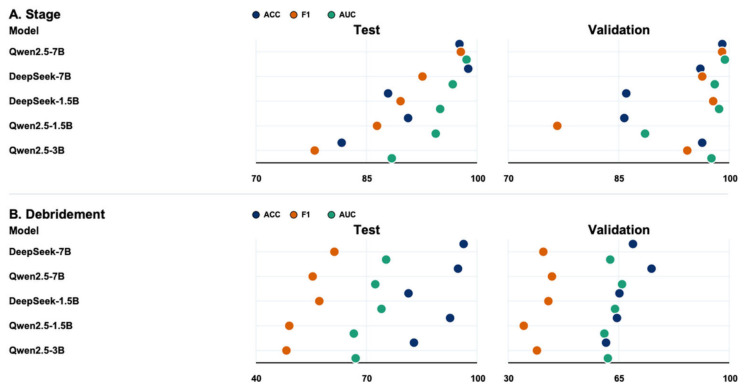
Left-right dot plots of LLM-only model performance for stage and debridement classification in the test and validation sets. Debridement values summarize Clean, Primary, and Secondary labels; ACC, F1, and AUC are shown.

**Figure 7:**
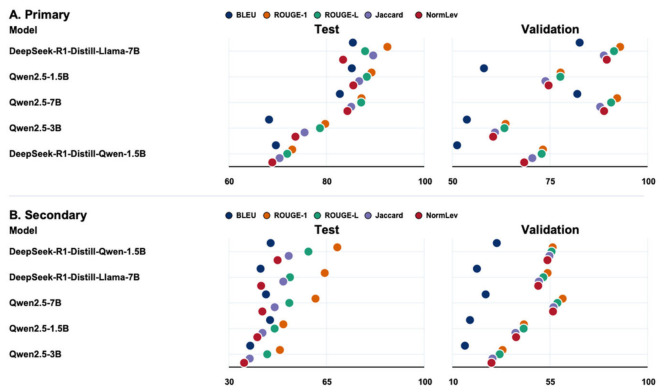
Left-right dot plots of LLM-only model performance for primary and secondary dressing generation in the test and validation sets. BLEU, ROUGE-1, ROUGE-L, Jaccard similarity, and NormLev are shown.

For the LLM-only models, the best-performing model varied across tasks and metrics (Tables S4 and S5), in Figure 6 and Figure 7. On the test set, DeepSeek-R1-Distill-Llama-7B achieved the highest accuracy for Stage classification (98.81% ± 1.06%) and the best overall performance for Debridement Intervention classification (92.86% ± 0.88% accuracy, 92.85% ± 0.88% F1-score, and 92.89% ± 0.89% AUC), whereas Qwen2.5-7B achieved the highest F1-score (97.80% ± 3.41%) and AUC (98.58% ± 2.17%) for Stage classification. On the validation set, Qwen2.5-7B showed the strongest overall classification performance, suggesting better generalizability than the conventional text encoders. For text generation (Table S5), in Figure 7, performance was task-dependent: DeepSeek-R1-Distill-Llama-7B performed best on Primary Dressing Choosing, whereas DeepSeek-R1-Distill-Qwen-1.5B performed best on Secondary Dressing Choosing. Notably, LLM-only models often showed high accuracy but relatively low F1-scores in dressing-related classification tasks, indicating substantial class imbalance. Overall, LLM-only models offered stronger semantic modeling and better validation-set classification performance, but their gains in complex dressing recommendation generation remained inconsistent.

### 4.4. Results of Image with Text Fusion Models

**Figure 8:**
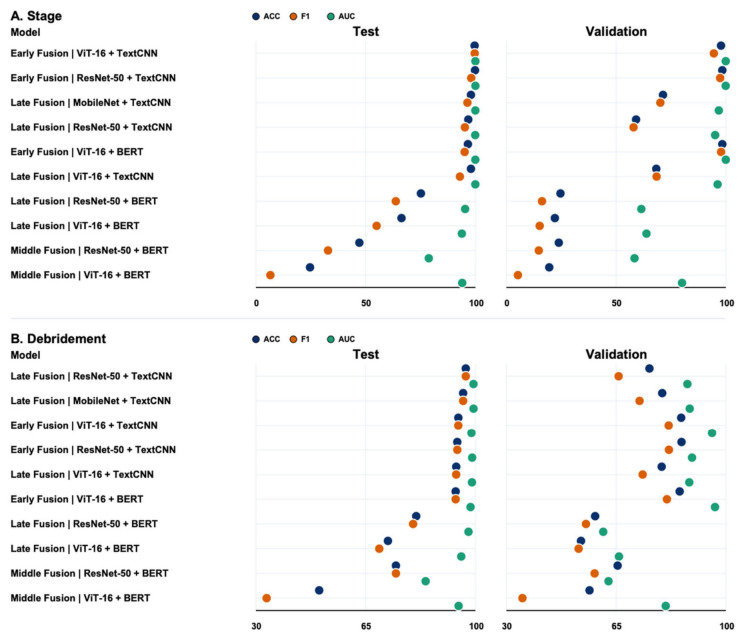
Left-right dot plots of image-text fusion model performance for stage and debridement classification in the test and validation sets. ACC, F1, and AUC are shown.

In the Image with Text fusion experiments, different fusion strategies showed clear task-dependent performance differences (Table S6), in Figure 8. For the classification tasks, early fusion achieved the best results for Stage classification, whereas late fusion was more advantageous for Debridement Intervention classification. On the test set, Early Fusion ResNet-50 with TextCNN achieved the highest Stage accuracy (99.83% ± 0.29%) and AUC (100.00% ± 0.00%), while Early Fusion ViT-16 with TextCNN achieved the highest F1-score (99.69% ± 0.27%). In contrast, for Debridement Intervention classification, Late Fusion ResNet-50 with TextCNN achieved the highest accuracy and F1-score (both 96.94% ± 1.35%), and Late Fusion MobileNet with TextCNN achieved the highest AUC (99.41% ± 0.29%). On the validation set, early fusion remained superior for Stage classification, with Early Fusion ViT-16 with BERT and Early Fusion ResNet-50 with TextCNN both reaching 98.38% accuracy, while Early Fusion ViT-16 with BERT achieved the highest F1-score (97.82% ± 1.24%) and AUC (99.97% ± 0.04%). For Debridement Intervention classification, the best-performing model varied by metric, but Late Fusion MobileNet with TextCNN achieved the highest accuracy (79.71% ± 1.06%). By contrast, middle fusion consistently underperformed on both datasets, with particularly poor Stage classification results, indicating that this strategy was not effective under the current setting. Overall, these findings suggest that the optimal fusion strategy differed by task, with early fusion being more suitable for Stage classification and late fusion being more robust for Debridement Intervention classification.

**Figure 9:**
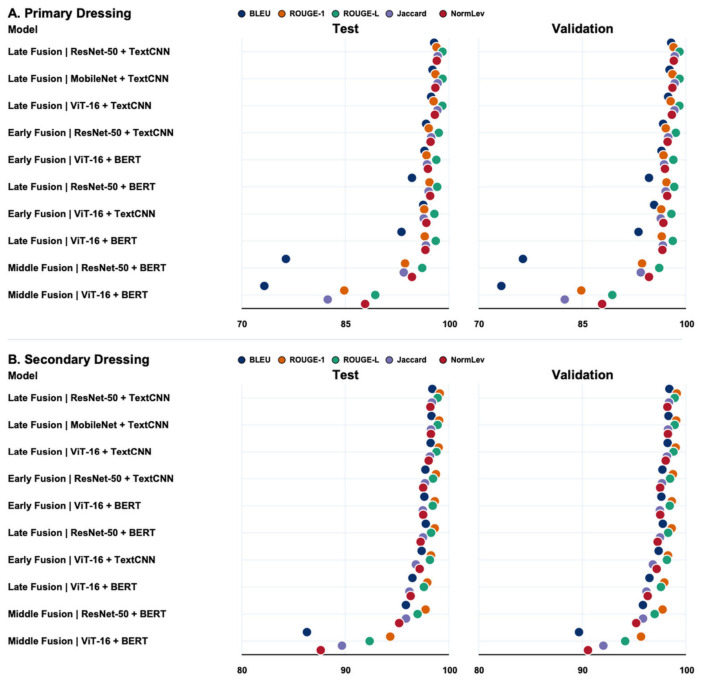
Left-right dot plots of image-text fusion model performance for primary and secondary dressing generation in the test and validation sets. BLEU, ROUGE-1, ROUGE-L, Jaccard similarity, and NormLev are shown.

For the text generation tasks, Image with Text fusion models substantially outperformed unimodal text models (Table S7), in Figure 9. On the test set, Late Fusion ResNet-50 with TextCNN achieved the best overall performance for both Primary Dressing Choosing and Secondary Dressing Choosing. For Primary Dressing Choosing, it achieved a BLEU score of 97.86% ± 0.45%, ROUGE-1 of 98.21% ± 0.26%, ROUGE-L of 99.07% ± 0.11%, Jaccard of 98.37% ± 0.16%, and Normalized Levenshtein of 98.25% ± 0.29%. For Secondary Dressing Choosing, the corresponding values were 98.40% ± 0.27%, 99.12% ± 0.11%, 98.91% ± 0.18%, 98.36% ± 0.22%, and 98.21% ± 0.30%. On the validation set, Early Fusion ResNet-50 with TextCNN showed the strongest overall performance, achieving the highest BLEU, ROUGE-1, ROUGE-L, and Jaccard scores across both dressing recommendation tasks, while Early Fusion ViT-16 with TextCNN achieved the highest Normalized Levenshtein. In contrast, middle fusion again showed substantially lower generation performance. Taken together, these results indicate that Image with Text fusion improved both classification and generation performance, with ResNet-50 with TextCNN emerging as the most stable conventional multimodal combination overall.

### 4.5. Results of Image with LLM Fusion Models

**Figure 10:**
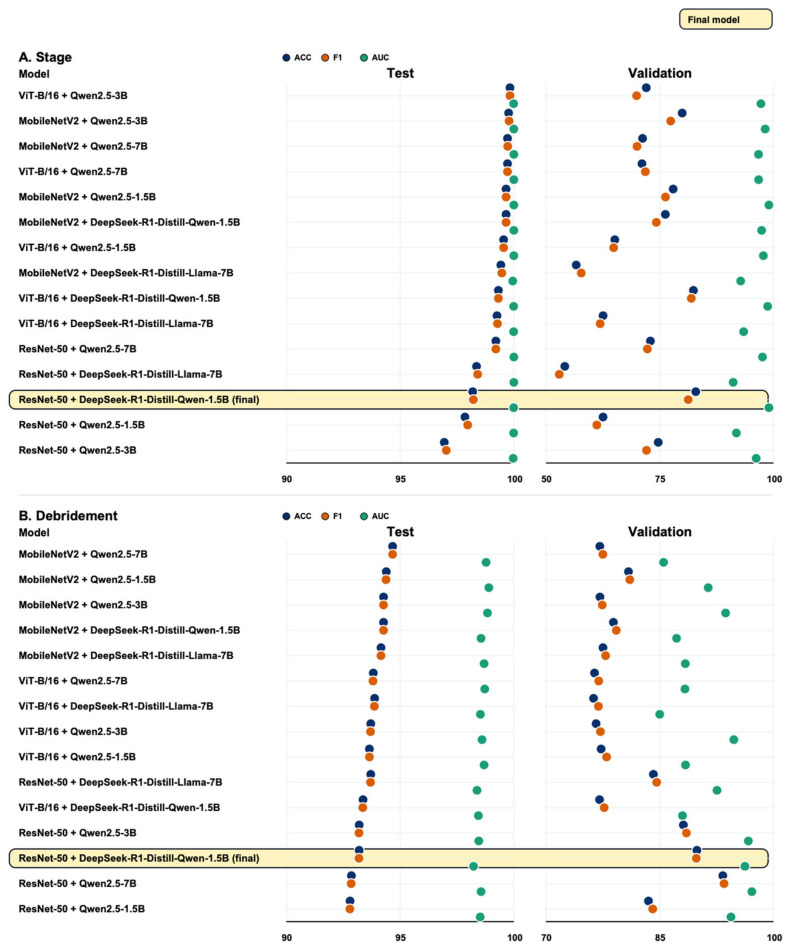
Left-right dot plots of image-LLM fusion model performance for stage and debridement classification in the test and validation sets. ACC, F1, and AUC are shown.

To investigate the contribution of large language models to multimodal clinical decision-making, three image encoders (ViT-B/16, ResNet-50, and MobileNetV2) were paired with five large language models, resulting in 15 Image-LLMs (Table S8), in Figure 10. For the classification tasks, Image-LLMs showed strong overall performance on the test set. For Stage classification, ViT-B/16 with Qwen2.5-3B achieved the best results, with an accuracy of 99.83% ± 0.26%, an F1-score of 99.83% ± 0.25%, and an AUC of 99.99% ± 0.02%. For Debridement Intervention classification, MobileNetV2 with Qwen2.5-7B achieved the highest accuracy and F1-score (both 94.67% ± 0.52%), while MobileNetV2 with Qwen2.5-1.5B achieved the highest AUC (98.90% ± 0.39%). Compared with the image-only and LLM-only baselines, incorporating visual information into LLM-based models consistently improved classification performance, indicating that wound images provided complementary clinical cues beyond text alone.

**Figure 11:**
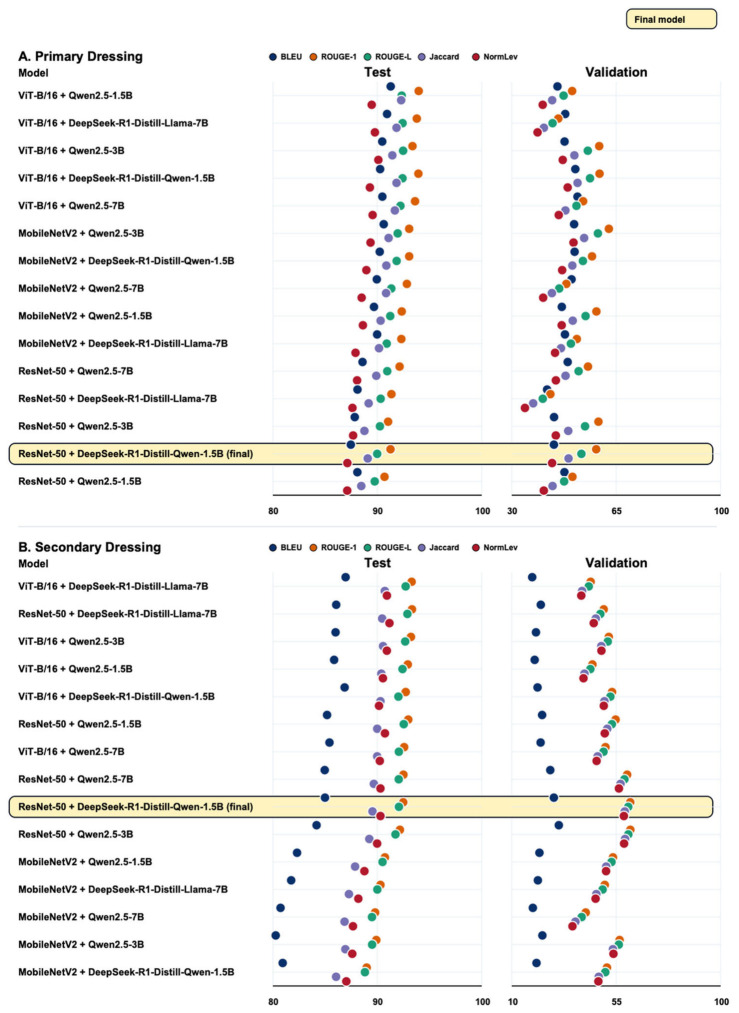
Left-right dot plots of image-LLM fusion model performance for primary and secondary dressing generation in the test and validation sets. BLEU, ROUGE-1, ROUGE-L, Jaccard similarity, and NormLev are shown.

Image-LLMs also performed strongly in text generation (Table S9), in Figure 11. For Primary Dressing Choosing, ViT-B/16 with Qwen2.5-1.5B achieved the best BLEU, ROUGE-1, and Jaccard scores (91.28% ± 0.74%, 93.96% ± 0.42%, and 92.36% ± 0.54%, respectively), whereas ViT-B/16 with Qwen2.5-3B achieved the best ROUGE-L and Normalized Levenshtein scores (92.47% ± 0.84% and 90.10% ± 0.86%). For the more challenging Secondary Dressing Choosing task, ViT-B/16 with DeepSeek-R1-Distill-Llama-7B achieved the highest BLEU and Jaccard scores (86.96% ± 0.55% and 88.31% ± 0.69%), while ResNet-50 with DeepSeek-R1-Distill-Llama-7B achieved the best ROUGE-1, ROUGE-L, and Normalized Levenshtein scores (93.32% ± 0.72%, 92.88% ± 0.65%, and 91.16% ± 0.69%, respectively). These results show that adding visual information preserved strong generation quality and improved performance in complex dressing recommendation tasks.

Because no single Image-LLM was optimal across all test-set metrics, model selection was based on overall validation performance. Among all candidates, ResNet-50 combined with DeepSeek-R1-Distill-Qwen-1.5B showed the most balanced performance. For stage classification, it achieved an accuracy of 82.92%, a macro-F1 score of 81.26%, and an AUC of 99.01%; for debridement intervention classification, the corresponding values were 89.91%, 89.83%, and 96.24%, respectively. It also maintained relatively stable generation performance, with BLEU/ROUGE-1/ROUGE-L scores of 44.13/58.30%/53.34% for Primary Dressing Choosing and 28.14/61.06%/60.20% for Secondary Dressing Choosing. Although this model did not rank first for every individual metric, it provided the best balance between classification performance, generation quality, and validation-set robustness, and was therefore selected as the optimal model for subsequent analyses.

To further evaluate the statistical support for final model selection, prespecified pairwise comparisons were performed between the final Image–LLM and representative baseline models using macro-F1 as the primary metric (Table S10). The final model showed nominally significant improvements in 6 of 10 comparisons. Specifically, it significantly outperformed the best image-only model in stage classification (*p* = 0.043) and the best conventional fusion model in debridement prediction (*p* = 0.028). More consistent advantages were observed in treatment recommendation tasks, where the final model significantly outperformed the best text-only and conventional fusion baselines for primary dressing recommendation (*p* = 0.016 and *p* = 0.011, respectively) and secondary dressing recommendation (*p* = 0.002 and *p* = 0.001, respectively).

### 4.6. Detailed Performance of the Optimal Model Across Classification Tasks

**Figure 12:**
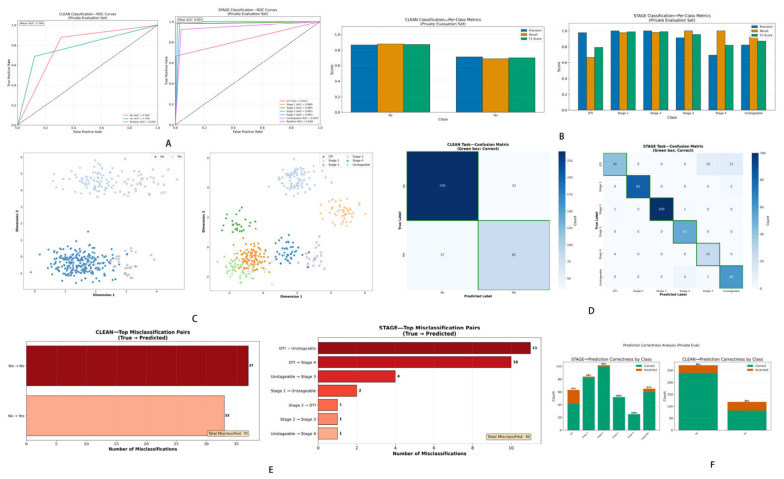
Detailed classification performance analysis of the optimal model. (**A**) The ROC curves for each class in the CLEAN and STAGE tasks on the private evaluation set; (**B**) Class-wise performance metrics for the two classification tasks on the private evaluation set, including precision, recall, and F1-score; (**C**) Dimensionality-reduced visualization of feature representations for the two classification tasks, with misclassified samples highlighted; (**D**) Confusion matrices for the two classification tasks. Green boxes indicate correctly classified regions; (**E**) Major misclassification pairs (true class → predicted class) and their frequencies; (**F**) Proportions of correctly and incorrectly classified samples for each class (The “X” marks in the figure indicate cases misclassified by the model).

As shown in Figure 3A, the optimal model demonstrated strong discriminative ability across most classes in the STAGE task, with AUC values ranging from 0.832 to 0.993. The highest AUC was observed for Stage 3 (0.993), whereas DTI showed the lowest AUC (0.832). In the CLEAN task, both No and Yes achieved an AUC of 0.784. The class-wise metrics further indicated that the model performed well across most categories in the STAGE task, although DTI showed relatively lower recall and Stage 4 showed relatively lower precision. In the CLEAN task, the model performed better overall for the No class than for the Yes class (Figure 12B).

The confusion matrices showed that the model correctly classified 321 of 391 samples in the CLEAN task, corresponding to an accuracy of 82.1%, and 361 of 391 samples in the STAGE task, corresponding to an accuracy of 92.3% (Figure 12D). In particular, the correct classification rates for Stage 3 and Stage 4 were both 100%, whereas DTI had the lowest correct classification rate at 66.7%. Misclassification analysis further showed that the main errors in the CLEAN task were Yes classified as No (37 cases) and No classified as Yes (33 cases). In the STAGE task, the dominant errors were DTI classified as Unstageable (11 cases) and DTI classified as Stage 4 (10 cases) (Figure 12E). The proportions of correct and incorrect predictions were consistent with these findings, indicating that although the optimal model achieved strong overall performance, DTI and the Yes class in the CLEAN task remained the main challenging categories (Figure 12F).

### 4.7. Interpretability Analysis of the Image and Text Modalities

**Figure 13:**
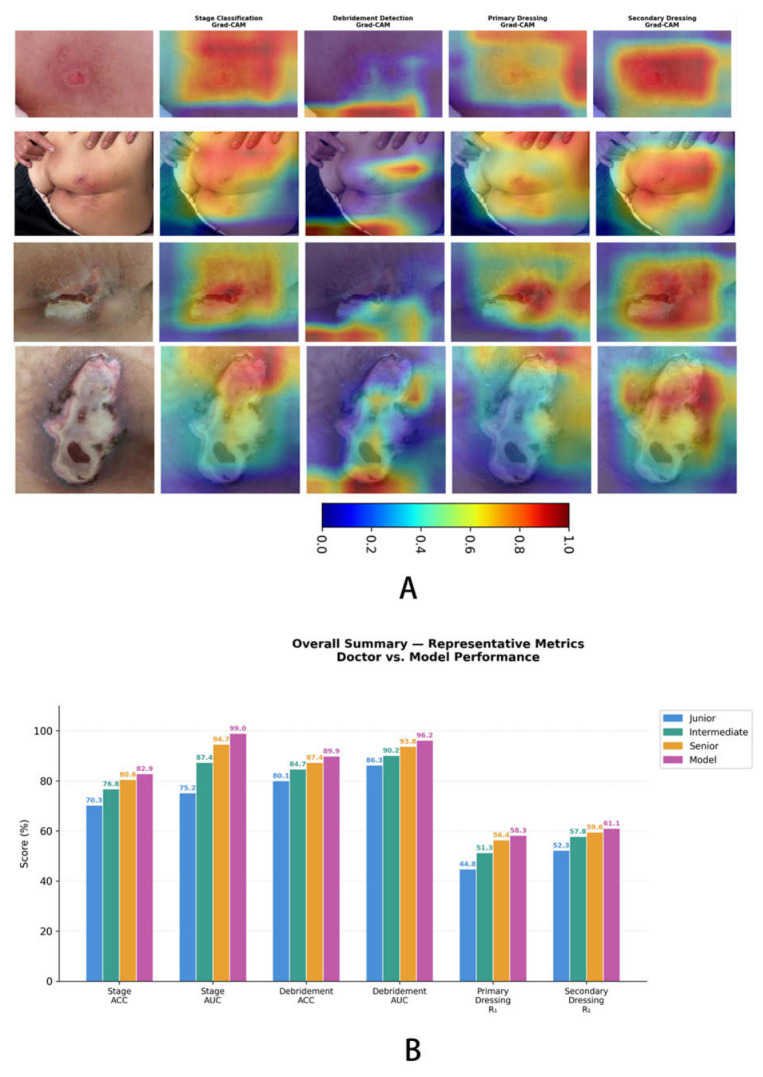
Image interpretability and expert comparison of the optimal model. (**A**) Grad-CAM-based image interpretability analysis showing the major regions attended by the model for stage classification, debridement assessment, and primary and secondary dressing recommendation tasks. (**B**) Performance comparison between the model and three specialist nurses with different levels of experience across representative metrics, including accuracy and AUC for stage classification and debridement assessment, as well as ROUGE-1 for primary and secondary dressing recommendation.

**Figure 14:**
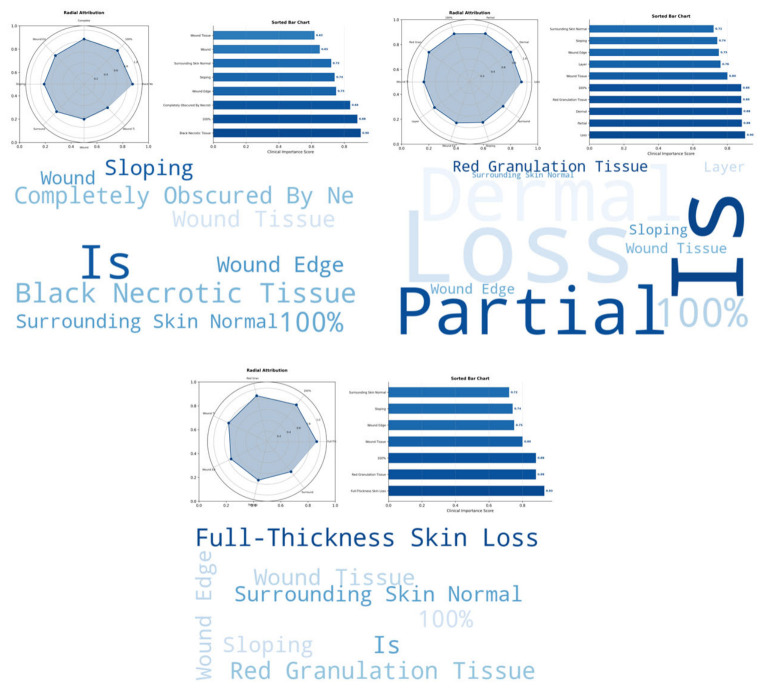
Text interpretability and expert comparison of the optimal model. Text interpretability analysis of representative cases, showing attribution results of high-contribution keywords using radar plots, ranked bar charts, and word clouds.

To examine the decision basis and clinical relevance of the optimal model, we performed multimodal interpretability analyses and compared its performance with specialist nurse assessments. Grad-CAM heatmaps showed that the image branch primarily attended to clinically meaningful regions, including the wound center, wound edge, necrotic or slough-covered areas, and the peri-wound transition zone (Figure 13A). For stage classification, attention was mainly localized to the wound margin and wound bed, whereas debridement prediction emphasized necrotic or treatment-relevant regions. By contrast, primary and secondary dressing recommendation showed broader attention patterns involving both the lesion core and surrounding skin, suggesting a greater reliance on global wound context. Text attribution analysis further showed that the model focused on clinically informative phrases, including “black necrotic tissue,” “red granulation tissue,” “partial loss,” “dermal loss,” “full-thickness skin loss,” “wound edge,” “surrounding skin normal,” “sloping,” and “100%” (Figure 14). These terms corresponded to key descriptors of tissue type, wound depth, wound-edge features, surrounding skin condition, and lesion extent, indicating that the model used textual information in a clinically targeted manner.

Comparison with three specialist nurses provided an additional clinical reference for model performance (Figure 13B). Model performance was assessed on the full validation set, whereas one junior, one intermediate, and one senior wound-care nurse independently evaluated a representative subset of 20 pressure injury photographs while blinded to model predictions. On the validation set, the final model achieved 82.9% accuracy and 92.9% AUC for stage classification, and 89.9% accuracy and 99.9% AUC for debridement prediction. In the nurse-evaluated subset, the three nurses achieved stage classification accuracies of 80.0%, 80.0%, and 75.0%, with corresponding AUCs of 90.0%, 90.0%, and 85.0%, respectively. For debridement assessment, their accuracy/AUC values were 75.0%/85.0%, 65.0%/75.0%, and 85.0%/95.0%, respectively. The model also achieved ROUGE-1 scores of 58.3% and 61.1% for primary and secondary dressing recommendation, respectively, which were higher than those observed in the nurse-evaluated subset.

Inter-expert agreement analysis further highlighted the variability of pressure injury treatment decisions (Supplementary Table S12). Complete agreement among all three nurses was observed in 65.0% of cases for stage classification and 55.0% for debridement assessment, but decreased to 25.0% and 35.0% for primary and secondary dressing recommendation, respectively. These findings suggest that the final model exhibited clinically interpretable decision patterns and strong validation performance when considered alongside specialist nurse assessments, supporting its potential as a standardized decision-support tool for pressure injury assessment and treatment recommendation.

## 5. Discussion

In this study, we adopted a stepwise modelling strategy, progressing from unimodal models to multimodal learning. After separately evaluating image-only and text-only models, we systematically compared conventional image–text fusion strategies and image–language model combinations in a unified multi-task setting. Considering classification performance, text-generation quality, and validation robustness, ResNet-50 combined with DeepSeek-R1-Distill-Qwen-1.5B was selected as the final model. This framework integrates visual wound features with language-based clinical information and jointly supports pressure injury stage classification, debridement necessity prediction, primary dressing recommendation, and secondary dressing recommendation. These findings suggest that multimodal learning better captures the complementary roles of wound photographs and clinical descriptions, providing a more clinically aligned approach to pressure injury assessment and treatment recommendation.

A In the image-only experiments, pretrained models consistently outperformed models trained from scratch, indicating that pressure injury image analysis still benefits from large-scale generic visual pretraining. This finding is consistent with previous wound image studies showing strong performance of ImageNet-pretrained convolutional backbones [8,11,22,26]. Although transformer-based visual models have shown promise in medical imaging, they did not consistently outperform CNN-based models in our setting. One possible explanation is that the current dataset size and real-world wound image variability, including differences in angle, illumination, background, and resolution, may favor CNN backbones that efficiently capture local lesion morphology, wound-edge structure, and tissue texture [27]. This trend was observed not only for stage classification but also for debridement intervention assessment.

The text-only and LLM-based experiments further showed that clinical wound descriptions contain substantial discriminative information. Structured wound descriptions can encode clinically meaningful features, such as tissue type, necrosis, exudate, wound edge, surrounding skin condition, and treatment needs, which are directly related to staging, debridement assessment, and dressing selection. Parameter-efficiently tuned or distilled large language models showed greater potential than conventional text encoders for complex semantic modeling and text generation. Similar advantages have been reported in other clinical NLP settings, where neural text encoders and LoRA-tuned LLMs have been used to extract clinically meaningful information from medical text and support prediction tasks [20,28]. Their strength appears to lie not only in more fluent language generation, but also in a greater ability to capture cross-sentence and cross-concept clinical semantics.

Nonetheless, the decline in performance observed for LLM-only models on more complex dressing-related tasks suggests that language models remain limited when direct visual context is unavailable. Text can encode high-level wound characteristics, but it cannot fully replace image evidence when decisions rely on directly observable features such as wound color, necrotic burden, tissue coverage, wound depth, and periwound condition. This limitation is consistent with the rationale for multimodal wound-care models, in which clinical text complements rather than replaces image-derived information [17,23]. Viewed together, these findings provide a strong rationale for multimodal fusion in pressure injury care.

The error patterns of the final model were clinically informative. Although performance was relatively stable across most categories, misclassifications were not randomly distributed. Instead, they were concentrated in categories with substantial visual overlap and semantic ambiguity, particularly deep tissue injury and unstageable pressure injury. In the private validation cohort, deep tissue injury showed the lowest area under the curve and recall among all stage categories, and the most frequent errors occurred among Stage 4, deep tissue injury, and unstageable lesions. This pattern is clinically plausible, as these categories may share several morphological features, including deep tissue involvement, necrotic coverage, uncertain wound depth, and poorly defined lesion boundaries. Similar findings have been reported in previous pressure injury prediction studies, in which deep tissue injury also showed lower precision than other stages [21,26].

The interpretability analyses further support this interpretation. Grad-CAM heatmaps showed that the model primarily focused on the wound center, wound edge, necrotic or slough-covered areas, and periwound transition zones, suggesting that image-based predictions were guided by clinically meaningful visual cues. Text attribution analysis showed that the model relied on terms such as “black necrotic tissue,” “red granulation tissue,” “full-thickness skin loss,” “wound edge,” and “surrounding skin normal,” all of which are directly relevant to stage assessment, debridement decisions, and dressing selection. Together, these findings suggest that the observed errors are more likely attributable to phenotypic overlap between clinically adjacent categories than to a lack of interpretable decision patterns.

This interpretation is also consistent with the specialist nurse assessment. Although the model was evaluated on the full validation set and the nurses assessed a representative subset, the comparison provided a useful clinical reference. The final model achieved strong validation performance, while the nurse assessments showed variability across tasks, particularly for dressing recommendation. These findings indicate that the model has potential as a standardized clinical decision-support tool, especially for improving consistency, efficiency, and traceability in complex wound-care workflows. However, its difficulties in borderline categories such as deep tissue injury and unstageable pressure injury also emphasize that the model should be viewed as an assistive tool rather than a replacement for expert clinical judgment.

For real-world deployment, several practical issues still need to be addressed. Because the LLM component may increase computational cost and inference latency, future implementation should consider model compression, quantization, local deployment, or secure server-based inference to ensure timely responses within bedside or outpatient wound-care workflows [29]. The system should also be integrated into existing wound documentation and electronic medical record systems, with outputs presented as interpretable decision-support suggestions rather than automatic treatment instructions [30]. In addition, wound photographs and clinical notes may contain sensitive patient information, requiring de-identification, encrypted storage, access control, and compliance with data-protection regulations [31]. Before clinical adoption, prospective multicenter validation, transparent reporting of intended use and limitations, post-deployment monitoring, and human-in-the-loop oversight will be necessary.

Several limitations should be acknowledged. First, the sample size remained limited, and some tasks were affected by class imbalance, which may have reduced robustness for minority classes and borderline cases. Second, because the public dataset was de-identified and the private evaluation cohort was retrospectively collected and anonymized, patient-level demographic information, standardized skin tone annotations, and detailed anatomical wound locations were not systematically available. This limited our ability to assess potential subgroup performance differences and algorithmic fairness across diverse patient populations. Third, the current model was restricted to wound images and text descriptions, without access to more comprehensive longitudinal clinical information, such as laboratory parameters, infection-related markers, nutritional status, and clearly defined follow-up time points. These factors can influence debridement and dressing decisions in routine practice. Fourth, although the study included a private evaluation cohort and comparison with human experts, it remained retrospective in design. Further validation in multicenter, prospective, and real-world workflow settings is therefore needed.

## 6. Conclusions

In this study, we proposed a multimodal multi-task framework that integrates wound images and clinical text to support pressure injury stage classification, debridement assessment, primary dressing recommendation, and secondary dressing recommendation. The final ResNet-50 + DeepSeek-R1-Distill-Qwen-1.5B model showed the best overall balance across classification performance, text-generation quality, and validation-set robustness, and interpretability and expert-comparison analyses further supported its clinical relevance. Nevertheless, the system should be regarded as an assistive decision-support tool rather than a replacement for expert judgment. Future work should focus on multicenter prospective validation, standardized collection of demographic and imaging metadata, incorporation of longitudinal clinical variables, lightweight deployment, integration into clinical workflows, and human-in-the-loop evaluation before real-world adoption.

## Data Availability

The datasets presented in this study are not currently publicly available because they are associated with an ongoing publication process. The datasets will be made openly available upon publication of this article at Zenodo: https://zenodo.org/records/18155016 (accessed on 1 January 2026).

## Supplementary Materials

The following supporting information can be downloaded at: https://www.mdpi.com/article/10.3390/bioengineering13060642/s1, Table S1: Image-only model performance for pressure injury stage classification and debridement intervention prediction; Table S2: Conventional text-only model performance for pressure injury stage classification and debridement intervention prediction; Table S3: Conventional text-only model performance for dressing recommendation generation; Table S4: LLM-only model performance for pressure injury classification; Table S5: LLM-only model performance for primary and secondary dressing recommendation generation; Table S6: Image-text fusion model performance for pressure injury stage classification and debridement intervention prediction; Table S7: Image-text fusion model performance for primary and secondary dressing recommendation generation; Table S8: Image-LLM fusion model performance for pressure injury stage classification and debridement intervention prediction; Table S9: Image-LLM fusion model performance for primary and secondary dressing recommendation generation; Table S10: Pairwise statistical comparisons between the final model and representative baselines; File S1: Prompt templates and LLM configuration details; Table S11: Representative pressure injury annotations and structured labels; Table S12: Three-expert agreement across classification and dressing recommendation tasks.

## Abbreviations

The following abbreviations are used in this manuscript:

PIs	Pressure injuries
AI	Artificial intelligence
CNN	Convolutional neural network
ViT	Vision Transformer
LLM	Large language model
LoRA	Low-Rank Adaptation
RYB	Red–Yellow–Black
BERT	Bidirectional Encoder Representations from Transformers
RNN	Recurrent neural network
LSTM	Long short-term memory
MLP	Multilayer perceptron
DTI	Deep tissue injury
ACC	Accuracy
AUC	Area Under The Receiver Operating Characteristic Curve
NormLev	normalized Levenshtein similarity
PISTA	Pressure Injury Staging and Treatment Annotation

## References

[1] Levine J.M., Delmore B. (2024). Pressure Injuries and Skin Failure. Clin. Geriatr. Med..

[2] Gillespie B.M., Walker R.M., Latimer S.L., Thalib L., Whitty J.A., McInnes E., Chaboyer W.P. (2020). Repositioning for pressure injury prevention in adults. Cochrane Database Syst. Rev..

[3] Sugathapala R., Latimer S., Balasuriya A., Chaboyer W., Thalib L., Gillespie B.M. (2023). Prevalence and incidence of pressure injuries among older people living in nursing homes: A systematic review and meta-analysis. Int. J. Nurs. Stud..

[4] Li Z., Lin F., Thalib L., Chaboyer W. (2020). Global prevalence and incidence of pressure injuries in hospitalised adult patients: A systematic review and meta-analysis. Int. J. Nurs. Stud..

[5] Padula W.V., Delarmente B.A. (2019). The national cost of hospital-acquired pressure injuries in the United States. Int. Wound J..

[6] Mervis J.S., Phillips T.J. (2019). Pressure ulcers: Prevention and management. J. Am. Acad. Dermatol..

[7] Lei C., Jiang Y., Xu K., Liu S., Cao H., Wang C. (2025). Convolutional Neural Network Models for Visual Classification of Pressure Ulcer Stages: Cross-Sectional Study. JMIR Med. Inform..

[8] Seo S., Kang J., Eom I.H., Song H., Park J.H., Lee Y., Lee H. (2023). Visual classification of pressure injury stages for nurses: A deep learning model applying modern convolutional neural networks. J. Adv. Nurs..

[9] Ikuta K., Fukuoka K., Kimura Y., Nakagaki M., Ohga M., Suyama Y., Morita M., Umeda R., Konishi M., Nishikawa H. (2024). An ingenious deep learning approach for pressure injury depth evaluation with limited data. J. Tissue Viability.

[10] Tusar M.H., Fayyazbakhsh F., Zendehdel N., Mochalin E., Melnychuk I., Gould L., Leu M.C. (2025). AI-Powered Image-Based Assessment of Pressure Injuries Using You Only Look once (YOLO) Version 8 Models. Adv. Wound Care.

[11] Demir Üçtepe A.S., Battal A.E., Güleç C., Ergün E., Bakcaci A., Karadağ A., Demir Üçtepe Ç.G. (2025). Artificial Intelligence-Enabled Staging Classification of Pressure Injuries. Adv. Skin Wound Care.

[12] Ge X., Du L., Zheng S., Shi A. (2025). Development and application of an intelligent pressure injury assessment system using AI image recognition. Technol. Health Care.

[13] Cho Y.B., Yoo H. (2025). Development of a pressure ulcer stage determination system for community healthcare providers using a vision transformer deep learning model. Medicine.

[14] Hu E.J., Shen Y., Wallis P., Allen-Zhu Z., Li Y., Wang S., Wang L., Chen W. Lora: Low-rank adaptation of large language models. Proceedings of the ICLR 2022.

[15] Dabas M., Schwartz D., Beeckman D., Gefen A. (2023). Application of Artificial Intelligence Methodologies to Chronic Wound Care and Management: A Scoping Review. Adv. Wound Care.

[16] Anisuzzaman D.M., Patel Y., Rostami B., Niezgoda J., Gopalakrishnan S., Yu Z. (2022). Multi-modal wound classification using wound image and location by deep neural network. Sci. Rep..

[17] Fard R.S., Agu E., Busaranuvong P., Kumar D., Gautam S., Tulu B., Strong D. (2025). Multimodal AI for Home Wound Patient Referral Decisions From Images With Specialist Annotations. IEEE J. Transl. Eng. Health Med..

[18] Mohan R., Raja N.S.M., Damasevicius R., Taniar D., Prabha S., Rajinikanth V. Vision transformer based diabetic foot-ulcer detection: A study. Proceedings of the 2024 Tenth International Conference on Bio Signals, Images, and Instrumentation (ICBSII).

[19] Sun D., Hadjiiski L., Gormley J., Chan H.P., Caoili E., Cohan R., Alva A., Bruno G., Mihalcea R., Zhou C. (2024). Outcome Prediction Using Multi-Modal Information: Integrating Large Language Model-Extracted Clinical Information and Image Analysis. Cancers.

[20] Jung H., Kim Y., Seo J., Choi H., Kim M., Han J., Kee G., Ko S., Kim B., Choi B. (2025). Clinical Assessment of Fine-Tuned Open-Source LLMs in Cardiology: From Progress Notes to Discharge Summary. J. Healthc. Inform. Res..

[21] Wang Z., Xu Y., Xia K., Dai Y., Xu X., Chen J. (2026). Construction and validation of a multi-function artificial intelligence-assisted system for pressure injury recognition. Front. Physiol..

[22] Kim J., Lee C., Choi S., Sung D.-I., Seo J., Lee Y.N., Lee J.H., Han E.J., Kim A.Y., Park H.S. (2023). Augmented decision-making in wound care: Evaluating the clinical utility of a deep-learning model for pressure injury staging. Int. J. Med. Inform..

[23] Nguyen H., Agu E., Tulu B., Strong D., Mombini H., Pedersen P., Lindsay C., Dunn R., Loretz L. (2020). Machine learning models for synthesizing actionable care decisions on lower extremity wounds. Smart Health.

[24] Velozo B.C., Hong M.V., Bernardo L.C., Novelli E Castro M.C., Contreras-Ruiz J., Abbade L.P.F. (2025). Pressure Injuries: Prevention, treatment, and complications–Part II. An. Bras. Dermatol..

[25] Kottner J., Cuddigan J., Carville K., Balzer K., Berlowitz D., Law S., Litchford M., Mitchell P., Moore Z., Pittman J. (2019). Prevention and treatment of pressure ulcers/injuries: The protocol for the second update of the international Clinical Practice Guideline 2019. J. Tissue Viability.

[26] Lai T.-Y., Chou Y.-J., Liu C.-Y., Chen C.-W., Lin C.-T., Wang W.-C., Hsu Y., Hsieh M.-L., Chang S.-S. (2026). Novel two-stage deep learning framework for automated pressure injury classification. BMJ Health Care Inform..

[27] Takahashi S., Sakaguchi Y., Kouno N., Takasawa K., Ishizu K., Akagi Y., Aoyama R., Teraya N., Bolatkan A., Shinkai N. (2024). Comparison of Vision Transformers and Convolutional Neural Networks in Medical Image Analysis: A Systematic Review. J. Med. Syst..

[28] Zhang D., Yin C., Zeng J., Yuan X., Zhang P. (2020). Combining structured and unstructured data for predictive models: A deep learning approach. BMC Med. Inform. Decis. Mak..

[29] USA Food and Drug Administration (2025). Good Machine Learning Practice for Medical Device Development: Guiding Principles.

[30] Shick A.A., Webber C.M., Kiarashi N., Weinberg J.P., Deoras A., Petrick N., Saha A., Diamond M.C. (2024). Transparency of artificial intelligence/machine learning-enabled medical devices. npj Digit. Med..

[31] World Health Organization (2021). Ethics and Governance of Artificial Intelligence for Health.

