# Supplementary material for: Integrating Wound Images and Clinical Text for Pressure Injury Assessment and Treatment Recommendation
Source: Bioengineering (Basel). 2026 May 29;13(6):642. doi: 10.3390/bioengineering13060642 (PMC13295290; doi:10.3390/bioengineering13060642)
Supplement: Supplementary file 1 [file bioengineering-13-00642-s001.zip › File S1 Prompt Templates and LLM Configuration Details.pdf]

1.Overview of LLM Methods

Our study employed LLM-based approaches across three experimental settings, summarized in Table S1.

Table S1. Overview of the Three LLM-based methods used in this study.

	Method	Model(s)	Task
1	Closed-source API call	DeepSeek-chat, Qwen-turbo, moonshot-v1-8k-vision-preview	Wound four-label classification (stage, debridement, primary dressing, secondary dressing)
2	LoRA fine-tuning	Qwen2.5-1.5B/3B/7B-Instruct; DeepSeek-R1-Distill-Qwen-1.5B/7B	Wound four-label classification
3	Generative dressing recommendation	Qwen2.5-1.5B-Instruct + LoRA	Dressing recommendation and classification

2. Prompt Templates

2.1 API-based Classification (DeepSeek, Kimi, Qwen)

System Prompt (English translation)

You are an expert in medical wound classification. Based on the following wound description, please determine its classification. Output strictly in the following JSON format and do not output anything else: {"stage": "...", "debridement": "...", "primary_dressing": "...", "secondary_dressing": "..."}. Candidate categories are as follows — stage: Stage 1, Stage 2, Stage 3, Stage 4, Unstageable, Deep Tissue Injury; debridement: Yes, No; primary dressing: [the full list of categories from the dataset]; secondary dressing: [the full list of categories from the dataset].

User Prompt

Wound description: [input wound description text]

Message Format

Messages are organized as role–content pairs: role="system" carries the system prompt; role="user" carries the user-supplied wound description.

2.2 Generative Dressing Recommendation (Qwen2.5 + LoRA)

System Prompt (English translation)

You are a professional wound-care specialist. Based on the wound description provided, please give accurate dressing recommendations.

User Prompt (English translation)

Based on the following wound description, please provide a professional dressing recommendation. Wound description: [input wound description text]. Please provide: (1) wound stage; (2) whether debridement is required; (3) primary dressing recommendation; (4) secondary dressing recommendation.

Expected Output Format

{"stage": "stage result", "debridement": "Yes/No", "primary_dressing": "recommended dressing", "secondary_dressing": "recommended dressing"}

2.3 LoRA Fine-tuned Classification (Qwen2.5, DeepSeek-R1-Distill)

System Prompt (shared by training and inference, English translation)

You are an expert in pressure-injury (pressure-ulcer) classification. Based on the wound description, predict the following four labels — stage: Stage 1, Stage 2, Stage 3, Stage 4, Unstageable, Deep Tissue Injury; clean (debridement): Yes, No; primary (primary dressing): hydrogel dressing, silver-ion dressing, alginate, foam dressing, hydrocolloid dressing, etc., or combinations such as "hydrogel dressing or silver-ion dressing or alginate"; secondary (secondary dressing): foam dressing, gauze/cotton pad, hydrocolloid dressing, none, or combinations such as "foam dressing or gauze, cotton pad". Output strictly in the following JSON format and include nothing else: {"stage": "...", "clean": "...", "primary": "...", "secondary": "..."}.

Full Prompt Format — Training (with labels)

[System prompt] Wound description: [input wound description text] [assistant] {"stage": "stage result", "clean": "Yes/No", "primary": "primary dressing", "secondary": "secondary dressing"}

Full Prompt Format — Inference (labels omitted, model completes)

[System prompt] Wound description: [input wound description text] [assistant]

3. Decoding Parameter Configuration

3.1 API Methods

Table S2. Decoding parameters for API-based methods.

Parameter	Wound Classification	Generative Recommendation	Notes
temperature	0.1	0.7	Low T for deterministic classification; higher T for diverse recommendation
top_p (nucleus)	Not used	0.9	Used only in generation
max_tokens	256	Not used	Used only in classification
top_k	Not used	Not used	Not configured
repetition_penalty	Not used	Not used	Not configured
presence_penalty	Not used	Not used	Not configured
frequency_penalty	Not used	Not used	Not configured
do_sample	Off (greedy)	On (sampling)	Sampling disabled in classification for reproducibility

3.2 LoRA Fine-tuned Models (HuggingFace Transformers)

Table S3. Decoding parameters for LoRA fine-tuned models.

Parameter	Value	Notes
max_new_tokens	512	Up to 512 new tokens are generated
temperature	0.7	Sampling temperature
top_p	0.9	Nucleus sampling probability threshold
do_sample	True (on)	Sampling-based generation enabled

4. Token Settings

Table S5. Token settings across experimental configurations.

Item	Classification Tasks	Generation Tasks
Max input context length	Variable (within model limit)	Variable (within model limit)
Max output tokens	256 (API) / 512 (LoRA)	512
Input truncation length	512 tokens (LoRA fine-tuning)	No truncation

The LoRA fine-tuning scripts uniformly enforce a maximum context length of 512 tokens for all models.

5. LoRA Hyperparameter Configuration

All LoRA fine-tuned models share the following hyperparameter configuration.

Table S6. LoRA fine-tuning hyperparameters.

Parameter	Value
LoRA rank (r)	8
LoRA alpha (α)	16
Target modules	q_proj, v_proj, k_proj, o_proj (Q/K/V/O attention projection layers)
LoRA dropout	Default value 0.1
Max sequence length	512 tokens
Training epochs	3 (default)
Learning rate	2×10^{-5} (default)
Batch size	3,5 (with gradient accumulation to simulate a larger effective batch)

6. Model Specifications

6.1 Closed-source API Models

Table S7. Closed-source API models.

Model Name	Provider	Use Case
deepseek-chat	DeepSeek AI	Wound classification
qwen-turbo	Alibaba Cloud Qwen	Wound classification

6.2 Open-source Fine-tuned Models

Table S8. Open-source models fine-tuned with LoRA.

Short Name	Full Model Name	Source	Parameters
qwen2.5-1.5b	Qwen/Qwen2.5-1.5B-Instruct	ModelScope	1.5B
qwen2.5-3b	Qwen/Qwen2.5-3B-Instruct	ModelScope	3B
qwen2.5-7b	Qwen/Qwen2.5-7B-Instruct	ModelScope	7B
deepseek-1.5b	deepseek-ai/DeepSeek-R1-Distill-Qwen-1.5B	ModelScope	1.5B
deepseek-7b	deepseek-ai/DeepSeek-R1-Distill-Qwen-7B	ModelScope	7B

7. Reproducibility

7.1 Environment and Dependencies

Table S9. Environment and dependencies.

Item	Detail
Model source	ModelScope (primary), HuggingFace (fallback)
Cache directory	/root/autodl-tmp/.cache/modelscope/models
Training framework	HuggingFace Trainer + peft (LoRA)
Training data format	JSONL (one sample per line)

7.2 Training Data Format

Each training sample contains a text field (the complete prompt) and a labels field (the four classification labels), stored in JSONL format.
